# Pharmacological, Toxicological, Phytochemical and Ethnobotanical Insights into North American *Aconitum* Species

**DOI:** 10.3390/molecules31172977

**Published:** 2026-08-25

**Authors:** Lily Kharlamb, Edward J. Kennelly

**Affiliations:** 1 PhD Program in Biochemistry, The Graduate Center, City University of New York, New York, NY 10016, USA; 2 Department of Biological Sciences, Lehman College, City University of New York, New York, NY 10468, USA

**Keywords:** *Aconitum columbianum*, *Aconitum delphiniifolium*, diterpenoid alkaloids, ethnobotany, toxicity

## Abstract

*Aconitum* L. species are widely recognized for their highly bioactive diterpenoid alkaloids, which contribute to their therapeutic potential and substantial toxicity. While Asian and European *Aconitum* species have been extensively studied in traditional medicine, phytochemistry, pharmacology, and toxicology, North American species remain comparatively underexplored. This review examines the ethnobotanical records, phytochemical data, toxicological mechanisms, and reported biological activities of North American *Aconitum* species, with emphasis on *A. delphiniifolium* and *A. columbianum*, the only native North American species with published alkaloid characterization. The literature was collected from scientific databases and ethnobotanical sources, with comparisons made to the better-studied Asian medicinal species, *A. carmichaelii*. Available research indicates that North American *Aconitum* species have limited documented medicinal use, although Alaskan Native groups historically used *Aconitum*-derived poisons in whale hunting. Phytochemical studies identified at least fifteen diterpenoid alkaloids in *A. delphiniifolium* and *A. columbianum*, including several compounds also found in *A. carmichaelii* with reported analgesic, anti-inflammatory, neuroprotective, and cytotoxic activities. However, direct pharmacological studies on North American species are lacking. These findings suggest that limited exploration of North American *Aconitum* reflects historical and research bias rather than the absence of therapeutic potential and highlights a significant research gap to support further metabolomic, pharmacological, and toxicological investigations of North American *Aconitum* species.

## 1. Introduction

*Aconitum* L. is a genus of flowering plants that belongs to the family Ranunculaceae. Around 400 species of *Aconitum* are distributed throughout the temperate regions of the Northern Hemisphere, including Europe, Asia, and North America [1]. The herbaceous perennials are sparsely distributed among the mountainous ranges of East and Southeastern Asia and Central Europe, with a smaller subset of species endemic to Northwest and Eastern United States [2]. There are seven known native North American *Aconitum* species, *A. columbianum* Nutt., *A. delphiniifolium* DC., *A. infectum* Greene, *A. maximum* Pall. ex DC., *A. noveboracense* A. Gray ex Coville, *A. reclinatum* A. Gray, and *A. uncinatum* L. [2]. Most *Aconitum* species produce a variety of natural secondary metabolites which are responsible for the well-documented physiological effects observed when used medicinally. The roots of the Asian species are used in traditional Chinese medicine (TCM) formulas to treat a variety of conditions. North American *Aconitum* species, though less studied than their Asian counterparts, hold significant therapeutic potential. Despite their presence in North America, the traditional uses and potential pharmacological applications of native *Aconitum* species remain poorly documented and scientifically under-explored. This review aims to provide a comprehensive examination of all available research on the pharmacology, phytochemistry, and ethnobotanical uses of the understudied North American species in the genus *Aconitum*.

Over 200 Asian *Aconitum* species have been documented for medicinal use, and *A. carmichaelii* alone has been the subject of hundreds of phytochemical and pharmacological studies. By contrast, of the seven *Aconitum* species native to North America, only two (*A. columbianum* and *A. delphiniifolium*) have undergone any phytochemical characterization, and no pharmacological studies have been conducted on any North American species. This disparity is striking given that the two characterized North American species share several bioactive alkaloids with *A. carmichaelii*, raising the question of whether the remaining five species may also produce pharmacologically relevant compounds. By evaluating existing studies and identifying key research gaps, this review seeks to offer a thorough understanding of how these North American *Aconitum* species compare to their better-studied Asian counterparts in terms of bioactive compounds, medicinal properties, and traditional uses. To our knowledge, no prior review has comprehensively compiled the available ethnobotanical, phytochemical, pharmacological, and toxicological data specifically for North American *Aconitum* species. This review addresses that gap by systematically evaluating the existing literature on all seven native North American species, comparing their known alkaloid profiles with those of the well-characterized Asian medicinal species *A. carmichaelii*, and identifying specific research priorities for future investigation.

## 2. Methodology

In-depth information on the *Aconitum* genus and North American *Aconitum* species was obtained via a literature search conducted for publications using various electronic databases, including Google Scholar, Scifinder, Web of Science Core Collection (1900-present), and Elsevier to locate primary and review research. Inclusion criteria: publications were included if they (1) reported on the taxonomy, distribution, morphology, ethnobotany, phytochemistry, pharmacology, or toxicology of any of the seven recognized North American *Aconitum* species; (2) described the phytochemistry or pharmacology of specific diterpenoid alkaloids that have been identified in North American species; or (3) provided comparative context from well-studied Asian (e.g., *A. carmichaelii*) or European *Aconitum* species relevant to understanding the North American taxa. Exclusion criteria included (1) publications not available in English nor with an English abstract or (2) conference abstracts without sufficient methodological detail. No date restrictions were applied. All titles and abstracts were screened for relevancy by both authors. Combinations of search terms used included names of all North American species, “*Aconitum columbianum*”, “*Aconitum delphiniifolium*”, “*Aconitum maximum*”, “*Aconitum reclinatum*”, “*Aconitum uncinatum*”, “*Aconitum noveboracense*”, and “*Aconitum infectum*”. Combination search terms were used, such as “*Aconitum delphiniifolium* and alkaloids”; “*Aconitum delphiniifolium* and ethnobotany”; “*Aconitum uncinatum* and phytochemistry”; and “*Aconitum maximum* and phytochemistry”. All titles and abstracts were screened for relevancy. All chemical structures found in North American *Aconitum* species were redrawn using ChemDraw (Version 23.1.2.7), based on published structural data and verified against the original literature.

Several websites and globally recognized online databases, including the Native American Ethnobotany Database, USDA Plant Profiles, were used for supplementary information. In addition, online resources like The World Flora Online and Plants of the World Online were consulted for geographical distribution and scientific name verification.

## 3. Distribution

The greatest concentration of species of *Aconitum* is found in Asia, with a smaller group in Europe. The seven known native North American *Aconitum* species (Table 1) extend from Alaska and Northwest Canada to the Southwest of the United States. Suitable habitats are usually at higher elevations along north–south mountain chains in both the Eastern and Western United States. The plants generally prefer riparian areas with moist but well-drained soil that retains cooler temperatures in partially shaded areas, including rocky outcrops, shaded cliffs and streamside sites within mountainous forests at higher elevations, but outlying populations are recorded in glacial refugia at lower altitudes [3].

**Figure 1:**
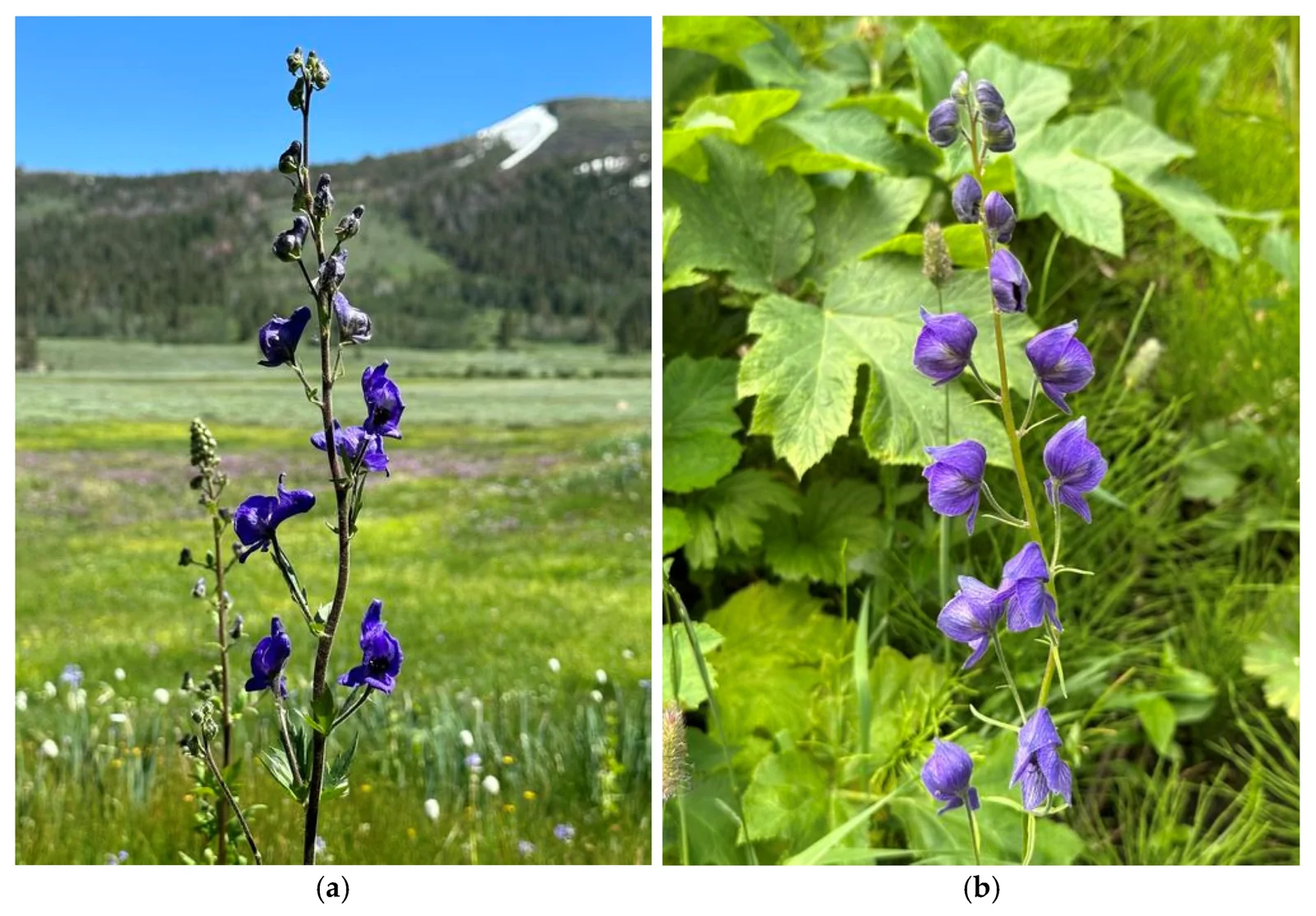
(**a**) *Aconitum columbianum* observed in Canada. Image source: GBIF.org, reprinted under a CC0 1.0 Public Domain Dedication license [5]. (**b**) *Aconitum delphiniifolium* observed in Canada. Image source: GBIF.org, reprinted under a CC0 1.0 Public Domain Dedication license [6].

There are several groups of tuberous *Aconitum* within the continental United States (excluding Alaska). *Aconitum columbianum* (Figure 1a) is the most widespread, naturally occurring in Western North America from British Columbia, Canada, through the Western United States down to Northern Mexico [2]. *Aconitum uncinatum*, also referred to as southern monkshood, occurs in the Eastern and Southeastern United States along the Appalachian Mountains from Pennsylvania to Georgia and Alabama. Smaller populations of the species *A. noveboracense*, known as Northern monkshood, occur in Iowa, New York, Ohio, and Wisconsin, [4]. Populations of *A. noveboracense* are sparse, isolated, and have significantly declined within the past century. *Aconitum reclinatum* grows in North Carolina, Pennsylvania, Tennessee, Virginia, and West Virginia. A very small declining population of species *A. infectum* Greene was recorded in the 1970s in Arizona. The other two North America species, *Aconitum delphiniifolium* and *Aconitum maximum*, are found much farther north and are the only two species of North American *Aconitum* to exist in sub-arctic climates. *Aconitum delphiniifolium* (Figure 1b) is distributed from the Russian far east to Alaska and Western Canada while *A. maximum* has a slightly smaller range from the Kamchatka Peninsula to Alaska and the Aleutian Islands. However, recent conservation assessments and population surveys are lacking for several North American *Aconitum* species, leaving their current population status and distribution uncertain.

**Table 1:** Seven North American *Aconitum* species, their native distribution, and conservation status from NatureServe.

North American Species	Native Distribution	Conservation Status	References
*Aconitum columbianum*	W. Canada to N. and W. Central U.S.A., Mexico (Sonora, Chihuahua); Arizona, British Columbia, California, Colorado, Idaho, Iowa, Mexico Northeast, Mexico Northwest, Montana, Nevada, New Mexico, New York, Ohio, Oregon, South Dakota, Utah, Washington, Wisconsin, Wyoming	Secure	[2,7]
*Aconitum delphiniifolium*	E. Siberia to W. Canada; Alaska, Alberta, Aleutian Is., British Columbia, Kamchatka, Khabarovsk, Magadan, Northwest Territories, Yakutskiya, Yukon	Secure	[7,8]
*Aconitum infectum*	Arizona	Critically Imperiled	[7,9]
*Aconitum maximum*	Kamchatka to SW. Alaska.; Alaska, Aleutian Is., Kuril Is.	Apparently Secure	[2,7]
*Aconitum noveboracense*	N. Central and NE. U.S.A. specifically Iowa, New York, Ohio, Wisconsin	Vulnerable–Federally threatened	[7,10,11]
*Aconitum reclinatum*	E. USA; North Carolina, Pennsylvania, Tennessee, Virginia, West Virginia	Vulnerable to Apparently Secure	[2,7]
*Aconitum uncinatum*	Illinois to E. U.S.A.	Apparently Secure	[2,7]

## 4. Morphology

*Aconitum* is a genus of herbaceous perennials characterized by alternate, palmately divided dark green leaves. The inflorescence takes the form of a long raceme, with bilateral flowers having five petals including a distinguishing hooded upper sepal. North American species vary in stature and growth habit. The plants generally stand 0.3 to 1.5 m tall and can feature a shorter erect stem or long, weaker, reclining, or twining stems, depending on the species and the environment in which they are growing. *Aconitum* species have flowers that are typically deep blue to violet, but some species can have white, greenish, or yellow flowers as well. *Aconitum* plants vary largely across the genus, and morphological intergradation is common.

Morphological differences among North American *Aconitum* species are notable and may have practical implications for identification and collection. *Aconitum columbianum*, the most widespread western species, typically produces erect stems 0.5–1.5 m tall with deep blue-violet flowers arranged in a terminal raceme [12]. In contrast, *A. uncinatum* of the Eastern United States has a characteristically weak, reclining stem that twines through surrounding vegetation, and its flowers are borne on more lax inflorescences [12]. *Aconitum uncinatum* can be distinguished from other North American species by its leaves, typically having three-to-five-lobed leaves, which are smaller compared with the three-to-seven-lobed leaves of other North American species. The foliar lobe dissections are much more shallow in *A. uncinatum* compared to the leaves of other North American species such as *A. noveboracense* and *A. delphiniifolium* [4,12]. *Aconitum reclinatum* is distinguished by its white to yellow flowers—unusual within the genus—and its trailing habit. *Aconitum noveboracense* closely resembles *A. columbianum* morphologically, but is distinguished by its restricted range and smaller population sizes [11]. The sub-arctic species *A. delphiniifolium* is generally shorter, slender, and erect (0.3–1.0 m). Its leaves are divided completely or almost completely to the base into three principal segments, which are further dissected into several more lobes which are narrower compared to the other species [12]. *Aconitum maximum* is the most robust of the North American species, with larger leaves and inflorescences. Its terminal inflorescences are frequently shortened rather than elongated as in other species. Root morphology provides additional diagnostic characters. *Aconitum columbianum* and *A. delphiniifolium* produce small tuberous roots measuring approximately 60 by 15 mm and 10–15 by 5 mm, respectively, usually with one contiguous daughter tuber [13]. The tubers of *A. maximum* are distally enlarged and measure approximately 20–50 by 5–20 mm [12]. *A. uncinatum* has 10–30 × 5–15 mm parent tubers which produce several daughter tubers separated by elongated connecting rhizomes, rather than the nearly contiguous tubers of *A. columbianum* and *A. delphiniifolium* [3]. *Aconitum reclinatum* is distinguished from other species by having very slender, fibrous root systems instead of tubers [4,12]. None of the North American species produce tubers as large as those of the Asian medicinal species such as *A. carmichaelii*.

## 5. Traditional Uses

Historically, a variety of *Aconitum* species have been used for medicinal purposes for thousands of years, due to the variety of biological effects imparted by the diterpenoid alkaloids produced by the plant, which can vary from relatively harmless to extremely toxic. Despite their relative toxicity, several alkaloids produced by *Aconitum* have shown significant therapeutic effects, which suggests that the less-studied American species may be useful medicinally [14,15].

### 5.1. Medicinal Uses

*Aconitum* species are known for their use in traditional medicine systems worldwide, most notably in TCM. The medicinal use of Asian *Aconitum* species, particularly *Aconitum carmichaelii and Aconitum kusnezoffi* Rchb., is well documented, and 76 of the more than 200 recorded Asian *Aconitum* species have been reported as medicinal herbs [16]. *Aconitum carmichaelii* and other species in the genus have been widely used across China, Japan, Korea, and Tibet for the treatment of pain, inflammation, cardiovascular conditions, and neurological disorders [14,17,18]. Among them, the processed lateral roots of *A. carmichaelii*, known as *Fuzi*, and processed mother roots, known as *Chuanwu* or *Radix Aconiti praeparata*, have been used in TCM for more than 2000 years [14,19,20].

Due to their high toxicity, *Aconitum* roots are used in TCM only after processing to reduce diester-diterpenoid alkaloid (DDA) content. According to regulations stipulated by the State Food and Drug Administration of China, only the detoxified, processed tubers and roots of *Aconitum* plants are allowed to be used in clinical decoctions, administered orally, and adopted as raw materials for pharmaceutical manufacturing [18]. Traditional processing methods, including soaking, boiling, steaming, roasting, or combinations thereof, hydrolyze the toxic DDAs into less toxic monoester or non-esterified derivatives [17]. The combination of these different processing methods results in multiple forms of *Fuzi*, which vary in toxicity and alkaloid profile [21]. In TCM, both processed lateral roots (*Fuzi*) and dried mother roots (*Chuanwu*, *Radix Aconiti praeparata*) are used in numerous formulations. Therefore, understanding the toxicity of North American *Aconitum* is critical prior to their development for any modern pharmacological uses.

Several isolated *Aconitum* alkaloids have been developed into single-entity drugs for pain management. Among these, lappaconitine is especially notable because it has been developed into a regulated pharmaceutical drug and is commonly used for postoperative and cancer-related pain management, particularly when NSAIDs or opioids are unsuitable [22,23]. In addition to lappaconitine, *Aconitum*-derived bulleyaconitine A has been used in China since 1985 for the treatment of chronic pain and does not appear to produce self-tolerance or cross-tolerance with morphine [24]. Therefore, we hypothesize that the underexplored American *Aconitum* species may also yield compounds useful as new single-entity drugs.

Traditional medicinal use has also been documented for European *Aconitum* species, particularly *Aconitum napellus*. This species is described in several 19^th^ century European *materia medica* publications, and homeopathic preparations, including topical salves, were used as a topical analgesic to treat neuralgia, gout, rheumatism, inflammation, and other painful conditions [25]. Collectively, these historical and modern applications demonstrate the therapeutic potential of the genus and provide a rationale for investigating the pharmacological properties of North American *Aconitum* species.

Although *Aconitum* species have been commonly used and prescribed by traditional medicine practitioners across many different cultures, due to its narrow therapeutic window and significant toxicity, improper use or dosage can and has resulted in many side effects including digestive distress, change in heart rate, and damage to the liver and kidneys. Accordingly, as research on North American *Aconitum* species advances, particular attention should be given to their toxicity and narrow therapeutic window.

### 5.2. Use as Poison

Although *Aconitum* alkaloids can have therapeutic effects after processing and at controlled doses, raw *Aconitum* species contain potent cardiotoxic and neurotoxic secondary metabolites, particularly DDAs, that can be lethal to humans and animals. Because of this toxicity, *Aconitum* has been widely recognized as a botanical poison and has historically been used in several regions for hunting, warfare, and intentional poisoning [26,27]. *Aconitum* was a common poison during Greco-Roman civilization, used by the Romans to kill enemies [25], so much so that eventually the emperor Trajan (98–117 AD) banned the growing of this plant in all Roman gardens [28]. One of the most widespread historical uses of *Aconitum* was as an arrow poison for hunting and warfare. *Aconitum*-derived poisons were used to coat arrows and other weapons by diverse cultures across Europe and Asia, including many indigenous peoples, with documented use in East Asia and China spanning at least 2500 years [29,30].

Many different species of *Aconitum* contain toxic alkaloids and have been used as a source of poison, including *Aconitum ferox* Wall., a Himalayan species associated with the traditional Indian poison known as bikh, and European *Aconitum* species, commonly known as wolfsbane, which were historically used as bait poisons for wolves and other predators [27,31,32]. Most *Aconitum* species are considered toxic to livestock. Ingestion can lead to severe neuromuscular and cardiac effects, muscle weakness, respiratory paralysis, and even sudden death. Even small amounts can be lethal, so *Aconitum* plants are very hazardous in grazing areas or pastures [33]. The widespread historical use of *Aconitum* as a poison demonstrates the powerful biological effects of its alkaloids and provides important context for evaluating both the risks and therapeutic potential of North American *Aconitum* species.

## 6. Ethnobotany of North American *Aconitum*

Despite the extensive medicinal and toxicological history of *Aconitum* in Asia and Europe, ethnobotanical records of North American species are far more limited. The most well-documented traditional use involves the preparation of *Aconitum*-derived arrow poisons by Indigenous peoples of Alaska and the Aleutian Islands. Aside from this practice, and a small number of reported medicinal applications, relatively little is known about the traditional uses of North American *Aconitum* species.

### 6.1. Arrow Poison by Alaskan Natives

The best-documented ethnobotanical use of North American *Aconitum* species is the preparation of arrow poison by Indigenous peoples of Alaska and the Aleutian Islands. *Aconitum* poison arrows were likely used by peoples native to Alaska and the surrounding islands, especially Kodiak Island and the Aleutian Islands, where species *A. delphiniifolium* and *A. maximum* grow [2].

Much of the evidence about *Aconitum*-poison arrows comes from research conducted by the renowned anthropologist Robert Heizer, who reported that the Aleuts and the Koniags used *Aconitum*-poisoned arrows for whale hunting [29]. There is no other evidence or record of the poison being used against people or in warfare by the Native Alaskans. The Aleuts believed that the use of poison would allow them to have a greater chance of inflicting a mortal wound on the animal when hunting. Anthropologist Lydia T. Black also reported that Aleuts used the poison to intoxicate the animal, impairing its motor control and thereby hastening its death [29]. The natives would aim to pierce the whale on its side closest to its fin, and then observe the wounded animal for a couple of days while they waited for it to succumb to the wounds before it washed or was towed ashore [34]. For whaling, the Aleuts and Koniag peoples used wooden lances with detachable notched arrows, often fashioned from bone, polished slate, or obsidian, which was preferred by the Koniag [35].

The only account of how the poison mixture was made comes from a detailed account of a late 1700s expedition to map uncharted territory in Alaska by the 18^th^-century explorer Martin Sauer. He wrote, “they also use poison to their arrows, and aconite is the drug adopted for this purpose. Selecting the roots of such plants- as grow alone, their roots are dried and pounded, or grated; water is poured upon them, and they are kept in a warm place until fermented: when in this state, the men anoint the points of their arrows, or lances, which makes the wound that may be inflicted mortal” [36]. Upon retrieval of the whale carcass, the tissue around the wound made with the poison-coated arrow was excised before the meat was distributed [35]. There is also a report of a ritual in which the hunter of the whale would consume a piece of flesh from the area where the arrow had penetrated, and if they felt no ill effect from consuming the flesh, distribution of the meat commenced [34].

The traditional use of *Aconitum*-based arrow poisons has been extensively documented in Eastern Asia. The Ainu people of northernmost Japan and the Russian Far East were known to employ *Aconitum* by using it to make a poisonous paste known as “surku”, used to coat weapons for bear hunting [37]. Heizer theorized that this complex hunting technology migrated eastward across the Aleutian Islands, linking Old and New World hunting practices. It is most likely that the use of *Aconitum* extracts as a weapon poison was transmitted inter-culturally from the Ainu peoples of the neighboring Kamchatka peninsula and Kurile Islands to Alaskan islanders. [35].

It is clear that the knowledge of the traditional use of this poison was not widespread through the Aleutian Islands, instead restricted to the whalers themselves. Ethnobotanist Anore Jones, the author of *Plants That We Eat: Nauriat Niginaqtuat*, recounts a conversation she had with an Aleut elder in the 1970s, in which he confirms the prior use of *Aconitum* as the source of the whaling arrow poison, and goes on to say the only man he knew of left alive who knew the traditional method of preparation of the poison died without sharing this knowledge because they thought that there were no people left who could responsibly hold this knowledge [38].

Overall, the use of *Aconitum*-based poison in North America appears to be best documented in the context of Aleut whaling, where preparation and use were restricted to a small hereditary group of Native Alaskan whalers. This knowledge was likely transmitted orally and remained closely guarded until the practice became obsolete. The limited surviving information, including the lack of widespread knowledge among Aleut communities, indicates that the technique remained restricted to a small hereditary group of specialized whalers rather than becoming general cultural knowledge. In other words, while the technology likely crossed between cultures (from Ainu to Aleut), it did not spread within Aleut culture beyond the whaling specialists who received it. Instead, the whaling method and the use of *Aconitum* poison appear to have been introduced together as a specialized practice associated specifically with whalers [35].

### 6.2. Other Medicinal/Folk Uses in North America

Despite the popularity of *Aconitum* in Asia and Europe as both a poison and medicine, there is little knowledge and documentation of the use of North American *Aconitum* species by native peoples living in North America. It is commonly grown as a garden ornamental in the United States. There are few reports of North American species that were used by natives as a medicinal plant. It is possible that *A. delphiniifolium* was used as a medicine for an unspecified purpose by people belonging to the Salishan tribes of the American and Canadian Pacific Northwest [39]. In the book *Plants and the Blackfoot*, the author reports the use of *Aconitum* species, possibly *A. columbianum*, by Great Plains Native Americans for fever, acute respiratory, and acute throat infection [40].

The use of *A. napellus* for medicinal purposes was popularized in Europe during the mid-18th century, especially after Viennese physician Anton von Stoerck wrote about his experiments with it, claiming that it was of great use for ailments such as pains in joints, gout, paralysis, intermittent fever, neuralgia, and chronic rheumatism [32]. Homeopathic practitioners and even physicians in the United States began to recognize *Aconitum* for its medicinal effects and began importing *A. napellus* to prescribe it to patients. “Aconite” was mentioned 36 times in the 1843 *United States Dispensatory*, focusing mainly on the European species *A. napellus* [41]. *Aconitum napellus* is also listed in the *United States Pharmacopeia* of 1850, included with recipes for producing alcoholic extracts and tinctures from the roots, as well as a tincture made from the leaves [42]. None of the seven North American *Aconitum* species have been mentioned in the *United States Pharmacopeia.* The use of *A. napellus* as an herbal drug started to decrease in the early 20^th^ century due to several cases of accidental but fatal poisonings [32]. The use of *Aconitum* extracts or alkaloids in modern medicine was mostly discontinued in North America by the early 1940s [3]. Many states began to list *Aconitum* as a scheduled poison and placed restrictions on its sale and distribution, which contributed to the decline of its use [43].

## 7. Phytochemistry

*Aconitum* species produce hundreds of different bioactive secondary metabolites, including flavonoids, alkaloids, and free fatty acids [44]. However, other than alkaloids, no other compound classes have been investigated in any North American *Aconitum* species. This review focuses primarily on diterpenoid alkaloids because they are the most pharmacologically and toxicologically significant metabolites in the genus, and are the only compound class for which published data exist from North American species. Hundreds of diterpenoid alkaloids and their derivatives have been identified in *Aconitum*, demonstrating substantial structural diversity and pharmacological potential [45,46]. These compounds are concentrated mainly in the roots and are responsible for many of the genus’s biological effects and much of its toxicity [47]. Diterpenoid alkaloids are complex natural products with varying structures and biological activities. Diterpenoid alkaloids at proper therapeutic dosages can have a broad range of biological activities such as analgesic, anti-inflammatory, antinociceptive, and antiarrhythmic properties—hence its use in traditional medicine [46]. The majority of the published phytochemical research conducted on the genus *Aconitum* focuses on *A. carmichaelii*, which is the most commonly used species for medicinal purposes and in TCM. The physiological effect of these potent alkaloids is evident even through casual contact with the unprocessed root. For example, in the *Chinese Pharmacopeia*, *Aconitum* is noted to cause a local tingling or numbing sensation when touched or tasted due to the presence of DDAs [14,48]. DDAs also give the herb its characteristic bitter and acrid taste. However, most of the analgesic alkaloids that are well-documented in Asian medicinal *Aconitum* species have not been reported in many North American varieties, likely due to limited research on these species. Historical sources, such as the work of the Lloyd brothers in the late 19th and early 20th centuries, provide some insight into the use and properties of North American *Aconitum* species [49]. The Lloyd brothers described many of the North American *Aconitum* species, including a plant they identified as *Aconitum fischeri*, which was later reclassified as *Aconitum columbianum.* They reported that *A. fischeri* (*A. columbianum*) has a bitter taste and numbs the tongue, much like its more well-known Asian counterparts, and claim that it must possess alkaloids that will prove to be of therapeutic value [49].

The diterpenoid skeletons of these alkaloids originate from four isoprene units, with heterocycles containing ethylamine, β-aminoethanol, or methylamine nitrogen derived from amination [50]. These compounds vary in ring arrangement, functional groups, and carbon number and are commonly divided into C_18_, C_19_, and C_20_ diterpenoid alkaloids. They may also be classified according to esterification and toxicity as highly toxic DDAs, less toxic monoester diterpenoid alkaloids (MDAs), and substantially less toxic non-esterified diterpenoid alkaloids (NDAs) [46]. The DDA/MDA/NDA classification reflects a well-established structure–toxicity relationship: the presence of both an acetyl ester at C-8 and a benzoyl ester at C-14 (as in aconitine, mesaconitine, and hypaconitine) confers high toxicity through persistent activation of voltage-gated sodium channels [51]. Hydrolysis of one or both ester groups progressively reduces toxicity by orders of magnitude. Although diterpenoid alkaloids are produced by all plants in the *Aconitum* genus, the relative abundance of DDAs, MDAs, and NDAs varies among species and contributes to differences in both toxicity and pharmacological activity [51]. C19 diterpenoid alkaloids represent the largest category of diterpene alkaloids and are the most studied compounds present in *Aconitum* species [46]. Biosynthetically, C_19_ diterpenoid alkaloids are derived from a geranylgeranyl diphosphate (GGPP) precursor through cyclization, oxidation, and amination steps. C19 DDAs containing an acetyl group at C-8 and a benzoyl ester at C-14, including aconitine, mesaconitine, and hypaconitine, are among the principal metabolites responsible for the toxicity of *Aconitum*, although they also contribute to its potent analgesic and cardiotonic effects [18]. In traditional medicine, *Aconitum* roots are generally processed before use to reduce the concentration of toxic alkaloids and the risk of poisoning. Heating and cooking promote hydrolysis of DDAs to MDAs, thereby lowering DDA content and producing a less toxic preparation [15,21].

Published research pertaining to the chemical and metabolomic characterizations of North American species is limited and dates back to the late 20th century. Existing studies on *A. columbianum* and *A. delphiniifolium* have identified at least 15 diterpenoid alkaloids using nuclear magnetic resonance and other spectrometric tools (Table 2). Boido et al. (1984) extracted the aerial parts of *A. columbianum* using ethanol, followed by acid–base solvent partitioning and column chromatography over alumina to isolate individual alkaloids [52]. These alkaloids were identified using infrared spectroscopy, ^1^H, and ^13^C nuclear magnetic resonance (NMR) spectroscopy. Pelletier et al. (1985) similarly employed ethanol extraction of *A. columbianum* plants with chromatographic separation and spectroscopic identification [53]. Aiyar et al. (1986) characterized the C_19_ diterpenoid alkaloids of *A. delphiniifolium* flowers, stems, leaves, and roots using analogous extraction and NMR-based identification methods [54]. It should be noted that these studies, conducted in the 1980s, employed chemical techniques that, while rigorous for their time, lacked the sensitivity and resolution of modern LC-MS/MS and high-field NMR methods. Consequently, the fifteen alkaloids identified likely represent only a fraction of the total alkaloid diversity present in these species. Among the alkaloids that have been identified in North American species, all belong to the MDA or NDA classes, which is consistent with their lower expected toxicity relative to DDAs. This includes several notable biologically active alkaloids, which are also known to be produced by the well-studied Asian medicinal species, *A. carmichaelii* [52,53,54].

## 8. Pharmacology and Toxicology

**Figure 2:**
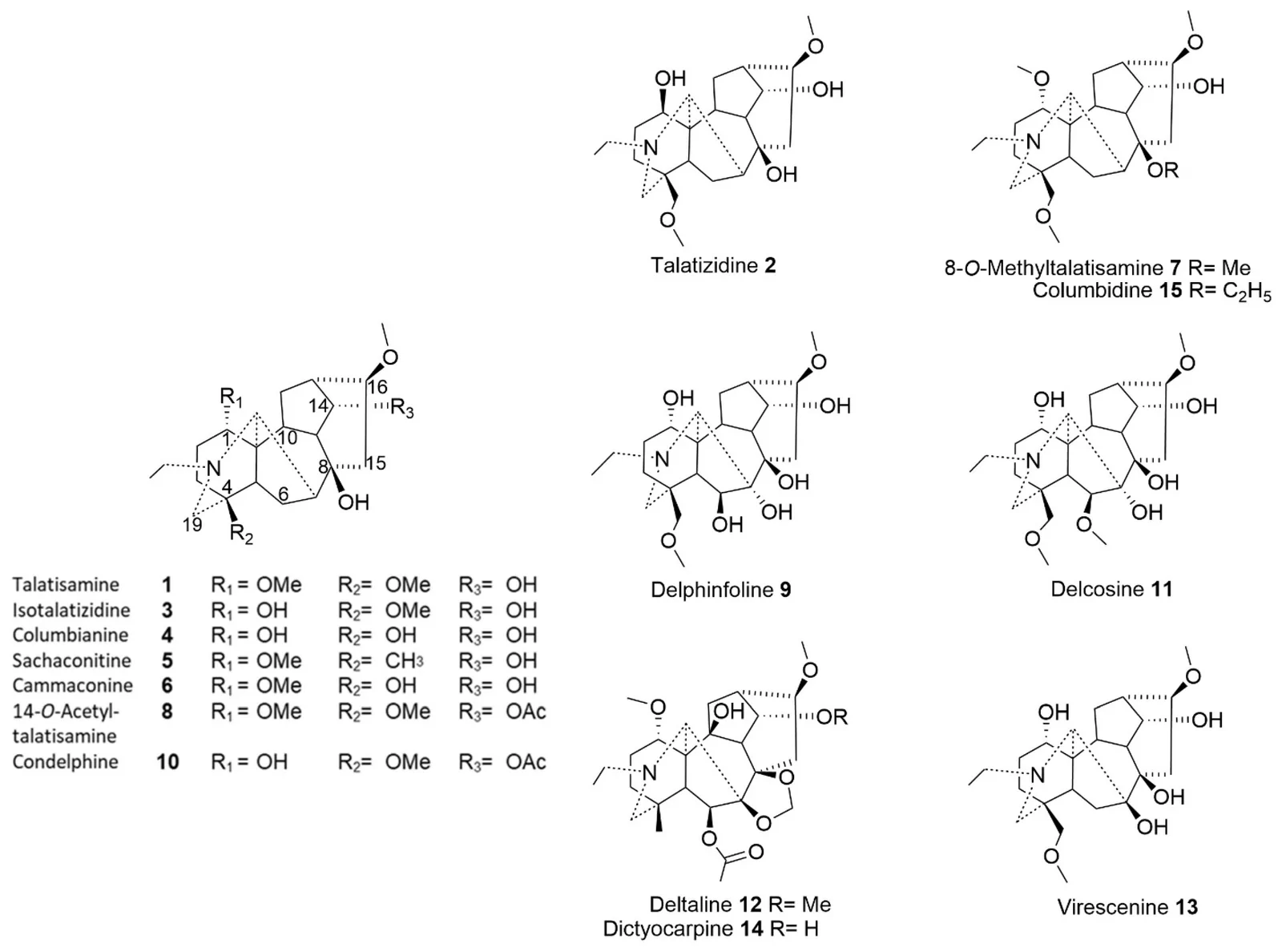
Chemical structures of fifteen diterpenoid alkaloids identified in the North American species *A. columbianum* and *A. delphiniifolium* [52,53,54].

The reported pharmacological activities of alkaloids identified in North American *Aconitum* species are summarized in Table 3. Of the fifteen diterpenoid alkaloids identified in *A. columbianum* and *A. delphiniifolium* (Figure 2, Table 2), five—talatisamine (**1**), talatizidine (**2**), isotalatizidine (**3**), 14-*O*-acetyltalatisamine (**8**), and condelphine (**10**)—are also present in the well-characterized Asian species *A. carmichaelii,* and have documented biological activities spanning analgesic, neuroprotective, anti-inflammatory, and cardiovascular effects. The pharmacological findings discussed below come from studies of alkaloids isolated from Asian species or tested as purified compounds in in vitro and in vivo animal models. No studies have directly tested extracts from any North American *Aconitum* species. The activities described below are therefore inferred based on shared alkaloid composition.

### 8.1. Mechanisms of Aconitum Toxicity

The well-known cardiotoxicity and neurotoxicity of *Aconitum* is attributed to DDAs such as aconitine, mesaconitine, hypaconitine, and derivatives which exert pharmacological effects via several mechanisms, including affecting noradrenaline reuptake, neuromuscular transmission, and voltage-gated sodium channels in nerves, cardiac tissue, and skeletal muscle. DDAs bind to open sodium channels at site 2, causing persistent sodium influx, arrhythmias, and possible heart failure [63]. Fatal poisoning from *Aconitum* most commonly results from refractory ventricular arrhythmias or asystolic cardiac arrest [63,64]. This mechanistic overview is provided for context, as these pathways are central to understanding *Aconitum* toxicity broadly. Notably, none of these highly toxic DDAs have been reported in North American species *A. columbianum* or *A. delphiniifolium* (Table 2), which instead contain MDAs and NDAs with lower toxicity profiles. Due to the lack in phytochemical studies on the north American species, their toxicity profile is unknown.

Despite the high toxicity risk from improper dosage, *Aconitum*, particularly Asian species, remains widely used in traditional medicine. The narrow therapeutic window of *Aconitum* alkaloids can limit their broader clinical applications. Various published works have also reported different biological activities of *Aconitum* fractions and extracts with respect to their active doses [14,65,66].

### 8.2. Mechanisms of Aconitum-Mediated Analgesia

*Aconitum* alkaloids exert powerful analgesic effects by interrupting pain transmission pathways and strongly suppressing inflammatory mediators [67]. The analgesic activity of *Aconitum* diterpenoid alkaloids operates through at least three distinct mechanisms, which differ by alkaloid class and have important implications for the therapeutic potential of compounds identified in North American species.

Although DDAs such as aconitine and mesaconitine have not been identified in North American species, their mechanism is briefly summarized here for comparative context. DDAs bind to site 2 of voltage-gated sodium channel α-subunits, causing persistent depolarization that suppresses pain signal transmission but is inseparable from cardiotoxicity, as reflected by extremely narrow LD_50_/ED_30_ ratios [68,69].

MDAs such as bulleyaconitine A and lappaconitine block voltage-gated sodium channels in a more selective manner, preferentially inhibiting hyperactive neurons while largely sparing normal neuronal activity [65]. This selectivity for overactive pain pathways is clinically relevant, particularly for chronic pain states. MDAs are significantly less toxic due to their less persistent interactions and lower affinity for binding sodium voltage gated-channels as compared to DDAs, which contributes to its improved safety profile while maintaining analgesic efficacy [69]. This also provides a mechanistic basis for the reduced toxicity observed after traditional processing of *Aconitum* roots [65].

In addition to modulating voltage-gated ion channels, several *Aconitum* alkaloids, including bulleyaconitine A and isotalatizidine (**3**), stimulate the opioid neuropeptide dynorphin A expression in spinal microglia through the cAMP–PKA–p38β–CREB signaling pathway [24,70,71]. The released dynorphin A activates presynaptic κ-opioid receptors on afferent neurons, producing antinociception [24]. This pathway is independent of direct sodium channel modulation and may therefore represent a mechanistically distinct route to analgesia.

These three mechanisms are not mutually exclusive; individual alkaloids may engage multiple pathways simultaneously. However, because no DDAs have yet been reported in North American *Aconitum* species (Table 2), the DDA-dependent sodium channel mechanism (first mechanism) is not directly applicable to these taxa. The clinical relevance for North American species lies instead in the MDA/NDA-associated mechanisms—particularly the dynorphin A/microglial pathway engaged by isotalatizidine (**3**), which has been identified in *Aconitum delphiniifolium* and *Aconitum columbianum*. The selective sodium channel blockade characteristic of MDAs offers potentially safer therapeutic profiles with wider margins between efficacy and toxicity.

### 8.3. Analgesic and Anti-Nociceptive Effects

Building on the mechanistic framework above, alkaloids shared between North American and Asian *Aconitum* species have demonstrated significant pain-relieving activity when studied as purified compounds isolated from Asian sources. While these compounds have been identified in North American species by phytochemical analysis, their analgesic activity has not been directly tested using North American plant material. Isotalatizidine (**3**), one of the many diterpenoid alkaloids in *Fuzi* that contributes to its well-known pain-relieving effects, is also present in the North America species. Isotalatizidine (**3**) produces dose-dependent analgesia in murine neuropathic pain models via the ERK1/2–CREB–dynorphin A pathway described above [72]. Other alkaloids such as talatisamine (**1**) exert analgesic effects through suppression of inflammatory cytokines like TNF-α at localized inflammatory sites [73]. However, even MDAs and NDAs with improved safety profiles relative to DDAs are not without risk, and the dose ranges at which therapeutic effects can be achieved without adverse cardiovascular or hepatic effects remain poorly defined for most of these compounds.

### 8.4. Neuroprotective Effects

The roots of *Aconitum*, like *Fuzi* and *Chuanwu*, are recognized for their neuropharmacological properties, such as antiepileptic, antidepressant, and antidementia actions [74]. Both talatisamine (**1**) and isotalatizidine (**3**), reported in the North American species show potential relevance to neurodegenerative disease. Talatisamine (**1**) is a potent potassium ion channel blocker, and potassium channel dysfunction has been implicated in Alzheimer’s and Parkinson’s disease [75]. In vitro, talatisamine (**1**) attenuates cytotoxicity induced by amyloid-β oligomers and reduces potassium ion loss-induced apoptosis in cultured cortical neurons [75]. Isotalatizidine (**3**) acts as a competitive inhibitor of both acetylcholinesterase and butyrylcholinesterase, a mechanism of action shared with currently approved Alzheimer’s therapeutics [76]. Condelphine (**10**), a C_19_ MDA, similarly inhibits cholinesterase activity, although through a non-competitive mechanism [77]. While these cholinesterase inhibition data for isotalatizidine (**3**) and condelphine (**10**) are promising, it should be noted that the studies were conducted using alkaloids isolated from *Delphinium denudatum* rather than from North American *Aconitum* species, and IC_50_ values and selectivity indices for these compounds have not been systematically compared with approved anticholinesterase Alzheimer’s therapeutics such as donepezil or rivastigmine.

### 8.5. Anti-Inflammatory Activities

Many of the C-19 diterpenoid alkaloids found in *Aconitum* species have documented anti-inflammatory effects. These compounds primarily target different key inflammatory pathways, making them subjects of interest in drug discovery for inflammatory and autoimmune conditions. Talatizidine (**2**) has demonstrated therapeutic potential for rheumatoid arthritis, possibly through regulation of the ALOX15B–PPARγ–PTGS2–FGF2–IL-1β–c-JUN–MMP13–TGF-β1 signaling axis [78,79]. Isotalatizidine (**3**) also exhibits anti-inflammatory, antirheumatic, and immunosuppressive properties [71,72]. Deltaline (**12**) has been used to develop novel anti-inflammatory derivatives. A diversity-oriented synthesis study created “deltanaline,” a derivative that demonstrated potent anti-inflammatory effects by inhibiting nuclear factor kappa-B (NF-κB)/mitogen-activated protein kinase (MAPK) signaling and inducing autophagy [80]. Talatisamine (**1**) was identified as one of the key compounds in modified *Fuzi* decoction that reduced TNF-α and monocyte chemotactic protein-1 in an in vivo osteoarthritis model, ultimately reducing inflammation via a TNF-α/TRAF2/NF-κB signaling-related anti-inflammatory mechanism [73].

### 8.6. Cytotoxicity

Studies of Asian *Aconitum* species and related diterpenoid alkaloids show that several C_19_ alkaloids can produce cytotoxic or antiproliferative effects against cancer cell lines, while potency, selectivity, and therapeutic relevance vary considerably [81,82,83]. Talatisamine (**1**), an alkaloid identified in both *A. columbianum* and *A. carmichaelii*, has been evaluated in an experimental glioblastoma model and showed cytotoxic effects against U-87 MG cells only at relatively high concentrations, with an LC_50_ of 2594 μg/mL, while also altering the expression of genes involved in cellular metabolism [84]. Additional findings from structurally related alkaloids provide further context for this activity class. 

Diterpenoid alkaloids with structural similarity to delcosine (**11**) have shown moderate to significant cytotoxicity against several human tumor cell lines, including A549 and H460 lung cancer cells, with reported IC_50_ values ranging from 7.97 to 28.42 micromolar [85]. Esterified derivatives of condelphine (**10**), which is produced by North American species *A. delphiniifolium*, and related diterpenoid alkaloids have also demonstrated increased selectivity against drug-resistant cancer cell lines, with some derivatives showing greater potency against vincristine-resistant cells than against non-resistant cells [85]. Several limitations should be considered when interpreting these findings. Talatisamine (**1**) showed cytotoxicity against U-87 MG cells only at a relatively high concentration (LC_50_ = 2594 µg/mL), which may limit its therapeutic relevance. In addition, most studies evaluated only a single cancer cell line and did not compare effects on normal and cancer cells. No in vivo tumor models have yet been used to assess alkaloids identified in North American species. Overall, these findings suggest that selected C_19_ diterpenoid alkaloids may have anticancer potential, but the available data remain insufficient to support conclusions about the anticancer activity of North American *Aconitum* species themselves.

**Table 3:** Reported biological activity of selected bioactive compounds identified in North American *Aconitum*.

Number	Compound	Reported Biological Activity	Reference
**1**	Talatisamine	Antiarrhythmic, analgesic, hypotensive, K+ ion channel blocker, anesthetic, cytotoxic, vasoprotective	[75,84,86,87]
**2**	Talatizidine	Anti-inflammatory, anti-rheumatic	[78,79]
**3**	Isotalatizidine	Analgesic, anti-nociceptive anti-inflammatory, anti-rheumatic, cytotoxic, cholinesterase inhibitor	[51,76,81]
**6**	Cammaconine	Cardiotonic	[88]
**8**	14-*O*-Acetyltalatisamine	Vasorelaxant. Ca^2+^ ion channel blocker	[87]
**10**	Condelphine	Cholinesterase inhibitor	[77,89]
**11**	Delcosine	Cytotoxic	[82]
**12**	Deltaline	Na^+^ channel blocker, analgesic, nicotinic acetylcholine receptor antagonist	[90,91]

This table includes only those compounds from Table 2 for which there is at least one published pharmacological study regarding biological activity. Compounds **4**, **5**, **7**, **9**, **13**, **14**, and **15** were excluded because no pharmacological data were found in the literature.

### 8.7. Knowledge Gaps and Comparison with Asian Species

While the alkaloids discussed above demonstrate a range of pharmacological activities, several critical limitations must be emphasized. All available pharmacological data were derived from purified compounds isolated from Asian *Aconitum* species or from the related genus *Delphinium* and evaluated either in vitro or in murine models. No pharmacological studies have directly examined extracts, fractions, or preparations from any North American *Aconitum* species. Phytochemical information is similarly limited. Only two of the seven North American species, *A. columbianum* and *A. delphiniifolium*, have undergone any phytochemical characterization, and that work dates to the 1980s using chemical methods that lacked the sensitivity of modern analytical chemical techniques. The remaining five species, *A. infectum, A. maximum*, *A. noveboracense*, *A. reclinatum*, and *A. uncinatum*, have neither been characterized phytochemically nor pharmacologically.

Even for the shared alkaloids identified in North American species, important pharmacological parameters remain undefined. Dose–response relationships, therapeutic indices, pharmacokinetic profiles, and organ-specific toxicity data are lacking for most compounds when studied individually and are entirely absent in the context of North American plant material. The assumption that alkaloids present in North American species will exhibit the same biological activities as when isolated from Asian species is reasonable but unvalidated, as alkaloid concentrations, ratios, and the presence of co-occurring metabolites (which may modulate activity through synergistic or antagonistic interactions) are likely to differ between species and populations.

These limitations also restrict meaningful comparison with the well-studied Asian medicinal species *A. carmichaelii*. While five of the fifteen alkaloids identified in North American species are shared with *A. carmichaelii*, the relative abundance of these compounds, the presence or absence of highly toxic DDAs (e.g., aconitine, mesaconitine, or hypaconitine), and the overall metabolomic profile of North American species remain unknown. Determining whether North American species produce DDAs—which have not been detected but also have not been systematically ruled out using modern methods—is a particularly important question, as it would directly affect both the therapeutic potential and safety profile of these plants.

A further gap concerns non-alkaloid metabolites. Flavonoids, polysaccharides, phenolic compounds, and fatty acids have not been systematically investigated in North American *Aconitum*, and the biological activities of crude extracts and fractions remain unknown. This is important because the effects of traditional *Aconitum* preparations may reflect interactions among multiple compound classes rather than the activity of individual alkaloids alone.

Future research should prioritize comprehensive untargeted metabolomic profiling of all seven North American species using LC-MS/MS and molecular networking; systematic bioactivity-guided fractionation of crude extracts; dose–response, safety pharmacology, and pharmacokinetic studies for key alkaloids; and comparative genomic/transcriptomic analysis to identify biosynthetic gene pathways and predict alkaloid production capacity across species.

## 9. Conclusions

North American *Aconitum* species remain substantially underexplored compared with their Asian and European counterparts, despite belonging to a genus known for producing highly bioactive diterpenoid alkaloids. The available literature indicates that native North American species have limited documented medicinal use, with the most detailed ethnobotanical records relating to the use of *Aconitum*-derived poisons in Alaskan whaling traditions. Phytochemical studies are also limited, with published alkaloid characterization available only for *A. columbianum* and *A. delphiniifolium*. These studies identified several bioactive C_19_ diterpenoid alkaloids, including talatisamine, talatizidine, isotalatizidine, 14-*O*-acetyltalatisamine, and condelphine, that are also reported from the well-studied Asian medicinal species *A. carmichaelii*. Reported activities of these shared or structurally related alkaloids include analgesic, anti-inflammatory, neuroprotective, cardiovascular, and cytotoxic effects, suggesting that North American *Aconitum* species may represent an overlooked source of pharmacologically relevant natural products.

Modern metabolomics and analytical platforms could substantially accelerate the characterization of these understudied species. Future research should prioritize comprehensive metabolomic profiling of all seven native North American species. Untargeted LC-MS/MS-based metabolomics, combined with molecular networking tools such as Global Natural Products Social Molecular Networking, would enable rapid identification of both known and potentially novel diterpenoid alkaloids across all seven North American species. Genome mining and transcriptomic analysis of biosynthetic gene clusters would help determine whether North American species contain the enzymatic pathways needed to produce DDAs or other alkaloid classes that have not yet been identified using conventional phytochemical methods. Alkaloid profiling of the understudied North American species should be followed by systematic evaluation of alkaloid toxicity, bioactivity, dose–response relationships, and pharmacokinetics. Structure–activity relationship (SAR) studies, guided by computational docking and QSAR modeling, could prioritize the most promising alkaloid scaffolds for further development as viable drug leads.

Given the rich ethnobotanical, phytochemical, and pharmacological literature on *Aconitum*, particularly in the context of traditional medicine, addressing this knowledge gap could enhance understanding of the therapeutic potential of this important genus. Shared alkaloid constituents, including isotalatizidine and talatisamine, support further investigation of North American *Aconitum* species as potential sources of pharmacologically active compounds; however, their safety and therapeutic efficacy remain to be experimentally established.

Conservation considerations are also relevant to future research on North American *Aconitum*. *Aconitum noveboracense* is federally listed as threatened under the U.S. Endangered Species Act, with sparse and declining populations in Iowa, Wisconsin, New York, and Ohio. *Aconitum infectum*, recorded only in Arizona in the 1970s, may be functionally extinct. Even more widespread species such as *A. reclinatum* and *A. uncinatum* occupy restricted habitats along the Appalachian Mountains that are vulnerable to habitat loss and climate change. Any future collection of North American *Aconitum* for phytochemical or pharmacological research must therefore be conducted in compliance with federal and state regulations, with appropriate collection permits, and using sustainable harvesting practices. Where possible, cultivation-based approaches, cell suspension cultures, or root tissue culture systems should be explored as alternatives to wild collection, particularly for rare or threatened species. Established ex situ propagation strategies, including tissue culture, greenhouse cultivation, and controlled field production, can provide reliable sources of plant material for scientific use while reducing pressure on wild populations [92].

North American *Aconitum* species could provide new avenues for the development of safer and more effective herbal treatments and lead to the discovery of novel therapeutic agents for treating pain, inflammation, neurological disorders, and cardiovascular conditions. Overall, the limited existing literature suggests that the lack of attention to North American *Aconitum* reflects a significant under investigated taxonomic and pharmacological domain rather than an absence of biological or therapeutic relevance.

## Figures and Tables

**Table 2:** Distribution of diterpenoid alkaloids identified in North American species *A. columbianum* and *A. delphiniifolium* and the Asian species *A. carmichaelii*.

Number	Compound	Molecular Formula	Identified in *A. columbianum*	Identified in *A. delphiniifolium*	Identified in *A. carmichaelii*	Reference
**1**	Talatisamine	C_24_H_39_NO_5_	✓	x	✓	[55]
**2**	Talatizidine	C_23_H_37_NO_5_	✓	x	✓	[56]
**3**	Isotalatizidine	C_23_H_37_NO_5_	✓	✓	✓	[54,56]
**4**	Columbianine	C_22_H_35_NO_5_	✓	x	x	[52]
**5**	Sachaconitine	C_23_H_37_NO_4_	✓	x	x	[57]
**6**	Cammaconine	C_23_H_37_NO_5_	✓	x	x	[55]
**7**	8-*O*-Methyltalatisamine	C_25_H_41_NO_5_	✓	x	x	[52]
**8**	14-*O*-Acetyltalatisamine	C_26_H_41_NO_6_	✓	x	✓	[58]
**9**	Delphinifoline	C_23_H_37_NO_7_	x	✓	x	[59]
**10**	Condelphine	C_25_H_39_NO_6_	x	✓	✓	[56]
**11**	Delcosine	C_24_H_39_NO_7_	x	✓	x	[60]
**12**	Deltaline	C_27_H_41_NO_8_	x	✓	x	[61]
**13**	Virescenine	C_23_H_37_NO_6_	x	✓	x	[62]
**14**	Dictyocarpine	C_26_H_39_NO_8_	✓	x	x	[53]
**15**	Columbidine	C_26_H_43_NO_5_	✓	x	x	[53]

Compounds identified in American species *A. delphiniifolium* and *A. columbianum*, [52,54]. ✓ = reported; x = not reported.

## Data Availability

No new data were created or analyzed in this study. Data sharing is not applicable to this article.

## Abbreviations

TCM—Traditional Chinese medicine; DDA—Diester-diterpenoid alkaloid; MDA—monoester diterpene alkaloids; NDAs—non-esterified diterpene alkaloids; NMR—nuclear magnetic resonance.
